# Supplementary material for: Rates and predictors of uptake of mental health support during the COVID-19 pandemic: an analysis of 26,720 adults in the UK in lockdown
Source: Soc Psychiatry Psychiatr Epidemiol. 2021 May 18;56(12):2287–97. doi: 10.1007/s00127-021-02105-w (PMC8129600; doi:10.1007/s00127-021-02105-w)
Supplement: Supplementary file 1 — Supplementary file1 (PDF 306 kb) [file 127_2021_2105_MOESM1_ESM.pdf]

Supplementary material

Table S1 Descriptive statistics (% or mean and standard deviation (SD)) before and after weighting

	Raw<br>%/Mean (SD)	Weighted<br>%/Mean (SD)
Age: 18-29	6.52%	19.47%
Age: 30-45	26.15%	26.06%
Age: 46-59	32.08%	24.14%
Age: 60+	35.25%	30.33%
Women (VS. men)	74.86%	50.68%
Ethnic minority (VS. white)	3.94%	12.78%
Education: low	12.84%	32.58%
Education: medium	16.73%	33.81%
Education: high	70.43%	33.61%
Employed (VS. Not employed)	62.08%	57.34%
Income <30k (VS. ≥30k)	38.74%	47.68%
Rural (VS. urban)	24.28%	20.58%
Living alone	21.04%	18.98%
Living with children	24.09%	24.36%
Living with others, no child	54.87%	56.66%
Close friends <3 (VS. ≥3)	24.76%	30.60%
Loneliness	4.79 (1.88)	4.98 (1.99)
Mental health diagnosis (VS. none)	17.24%	20.19%
Depression (PHQ-9)	6.16 (5.56)	6.85 (6.18)
Anxiety (GAD-7)	4.70 (4.96)	5.15 (5.44)
Coping: problem-focused	0.09 (0.50)	0.01 (0.50)
Coping: emotion-focused	0.10 (0.65)	0.01 (0.66)
Coping: avoidant	0.01 (0.52)	0.04 (0.55)
Coping: socially-supported	0.13 (0.67)	0.02 (0.69)
Personality: openness to Experience	0.08 (0.58)	0.00 (0.58)
Personality: conscientiousness	0.07 (0.69)	-0.01 (0.71)
Personality: extraversion	0.06 (0.70)	0.00 (0.71)
Personality: agreeableness	0.01 (0.36)	-0.01 (0.37)
Personality: neuroticism	-0.05 (0.67)	0.00 (0.70)

Table S2 Percentages of people using each strategy by characteristic groups (weighted)

	MED[1]	MH[2]	GP[3]	HL[4]	SFC[5]	FAM[6]
Age: 18-29	21.7%	17.9%	12.6%	14.6%	65.3%	69.1%
Age: 30-45	22.4%	10.3%	8.8%	10.7%	53.0%	55.5%
Age: 46-59	23.8%	6.8%	7.5%	7.3%	40.3%	41.0%
Age: 60+	13.3%	2.2%	3.2%	3.1%	22.4%	22.5%
Male	15.9%	6.1%	6.1%	6.4%	30.3%	33.1%
Female	23.7%	10.7%	8.9%	10.1%	55.4%	55.9%
White	20.4%	8.1%	7.3%	7.5%	41.7%	43.7%
Ethnic minority	16.2%	11.3%	8.7%	13.5%	52.1%	51.1%
Education: low	22.7%	5.9%	6.8%	5.8%	28.3%	32.2%
Education: medium	21.6%	9.7%	9.6%	10.6%	43.3%	46.5%
Education: high	15.3%	9.8%	6.1%	8.4%	57.0%	54.9%
Not employed	23.4%	9.4%	9.4%	9.3%	38.0%	38.6%
Employed	17.2%	7.8%	6.1%	7.6%	46.7%	49.2%
Income ≥30k	14.3%	7.5%	5.7%	6.9%	45.5%	47.0%
Income <30k	25.9%	9.6%	9.6%	9.9%	40.3%	42.1%
Urban	19.9%	8.8%	7.8%	8.7%	44.0%	46.2%
Rural	19.6%	7.4%	6.3%	6.7%	39.1%	38.8%
Living alone	24.3%	10.4%	9.0%	8.9%	41.3%	42.4%
Living with children	21.0%	8.4%	8.4%	9.5%	44.9%	50.2%
Living with others, no child	17.9%	7.9%	6.6%	7.6%	42.8%	43.0%
Close friend <3	17.2%	7.9%	6.8%	7.9%	44.9%	47.5%
Close friend ≥3	25.9%	9.8%	9.2%	9.2%	38.8%	38.2%
Loneliness <6	12.9%	4.1%	3.6%	4.0%	36.6%	38.2%
Loneliness ≥6	31.2%	15.7%	14.0%	15.4%	53.7%	55.3%
No mental health diagnosis	7.0%	3.9%	3.6%	4.4%	37.4%	38.3%
Mental health diagnosis	70.7%	26.4%	22.9%	23.6%	65.4%	69.8%
PHQ <10	11.8%	4.3%	3.3%	4.0%	38.1%	37.3%
PHQ ≥10	40.5%	19.1%	18.3%	19.4%	55.7%	63.7%
GAD <10	14.3%	4.9%	4.0%	4.8%	38.8%	38.8%
GAD ≥10	42.5%	23.2%	22.0%	22.9%	60.3%	68.6%
Coping: problem-focused (low)†	19.0%	6.9%	6.2%	6.1%	31.0%	34.6%
Coping: problem-focused (high)†	20.8%	10.4%	9.1%	10.9%	57.2%	56.5%
Coping: emotion-focused (low)†	19.5%	7.6%	7.7%	7.4%	33.6%	37.7%
Coping: emotion-focused (high)†	20.2%	9.6%	7.3%	9.5%	55.0%	53.5%
Coping: avoidant (low)†	12.4%	4.5%	3.7%	4.2%	35.1%	34.5%
Coping: avoidant (high)†	26.4%	12.0%	10.9%	11.9%	50.0%	53.6%
Coping: socially-supported (low)†	16.4%	4.1%	4.7%	4.8%	29.0%	25.6%
Coping: socially-supported (high)†	24.4%	14.2%	11.3%	12.9%	61.5%	69.7%
Personality: openness to Experience (low)†	19.0%	6.9%	6.7%	6.6%	33.9%	37.6%
Personality: openness to Experience (high)†	20.9%	10.5%	8.5%	10.6%	54.9%	53.9%
Personality: conscientiousness (low)†	21.3%	9.7%	8.0%	8.8%	42.2%	44.4%
Personality: conscientiousness (high)†	18.0%	6.9%	7.0%	7.7%	44.0%	44.9%
Personality: extraversion (low)†	23.3%	9.3%	8.0%	8.7%	42.1%	42.0%
Personality: extraversion (high)†	16.0%	7.5%	7.0%	7.9%	44.1%	47.6%
Personality: agreeableness (low)†	18.6%	8.1%	7.3%	7.3%	38.9%	39.9%
Personality: agreeableness (high)†	21.2%	8.9%	7.8%	9.4%	47.7%	50.1%

Personality: neuroticism (low)†	10.3%	3.5%	3.3%	3.5%	33.3%	33.0%
Personality: neuroticism (high)†	28.3%	12.8%	11.2%	12.5%	51.6%	55.0%

Notes: 1 Taking medication, 2 Speaking to mental health professionals, 3 Speaking to a GP or other health professionals, 4 Using helpline or other online services, 5 Self-care 6 Talking to family or friends; † Variables dichotomised at median.

Table S3 Frequencies of specific mental health conditions included in the alternative mental health diagnoses measure

Diagnosis	N
Schizophrenia	21
Bipolar disorder	148
Manic depression	48
Obsessive-compulsive disorder	215
Post-traumatic stress disorder	512
Eating disorder	222
Psychosis	31
Panic disorder	290
Anxiety	3,370
Depression	3,112
Other mental health condition	514
Total number of conditions	8,483
Total number of participants	4,864

Table S4 Cross-tabulation of two mental health measures

Mental health diagnosis (original)	Mental health diagnosis (alternative)		
	No	Yes	Total
No	16,160	1,699	17,859
	90.5%	9.5%	83.6%
Yes	344	3,165	3,509
	9.8%	90.2%	16.4%
Total	16,504	4,864	21,368
	77.2%	22.8%	100.0%

Table S5. Results from logit model on each strategy to support mental health (using alternative mental health diagnosis measure)

	Model I: Medication		Model II: Mental health professionals		Model III: GP/other health professionals		Model IV: Helpline/online services		Model V: Self-care/help		Model VI: family/friends	
	OR	95% CI	OR	95% CI	OR	95% CI	OR	95% CI	OR	95% CI	OR	95% CI
30-45 (VS. 18-29)	**2.26**	**[1.55,3.30]**	0.76	[0.53,1.10]	1.08	[0.72,1.63]	1.21	[0.83,1.75]	0.83	[0.63,1.09]	0.80	[0.58,1.09]
46-59 (VS. 18-29)	**2.84**	**[1.95,4.14]**	**0.64**	**[0.44,0.91]**	0.97	[0.65,1.45]	1.04	[0.74,1.46]	**0.60**	**[0.46,0.78]**	**0.54**	**[0.40,0.74]**
60+ (VS. 18-29)	**2.52**	**[1.68,3.77]**	**0.31**	**[0.20,0.48]**	0.75	[0.48,1.16]	0.71	[0.49,1.03]	**0.31**	**[0.23,0.41]**	**0.31**	**[0.22,0.42]**
Women (VS. men)	1.09	[0.87,1.37]	0.95	[0.68,1.31]	0.88	[0.65,1.18]	0.82	[0.62,1.08]	**1.96**	**[1.71,2.26]**	**1.41**	**[1.22,1.62]**
Ethnic minority (VS. white)	0.63	[0.39,1.00]	1.18	[0.69,2.01]	1.01	[0.65,1.59]	1.43	[0.94,2.18]	1.14	[0.82,1.59]	1.11	[0.83,1.50]
Education medium (VS. low)	0.80	[0.64,1.02]	1.16	[0.80,1.69]	1.02	[0.74,1.40]	**1.45**	**[1.05,1.99]**	**1.37**	**[1.13,1.66]**	**1.32**	**[1.09,1.61]**
Education high (VS. low)	**0.78**	**[0.62,0.99]**	**1.67**	**[1.19,2.34]**	0.94	[0.68,1.29]	**1.56**	**[1.15,2.11]**	**2.03**	**[1.67,2.47]**	**1.50**	**[1.24,1.82]**
Employed (VS. Not employed)	**0.78**	**[0.63,0.96]**	0.89	[0.66,1.19]	0.82	[0.61,1.11]	1.06	[0.81,1.37]	1.01	[0.86,1.18]	**1.26**	**[1.06,1.49]**
Income <30k (VS. ≥30k)	1.05	[0.85,1.30]	0.77	[0.58,1.02]	1.08	[0.80,1.46]	1.18	[0.91,1.52]	1.00	[0.85,1.17]	0.99	[0.84,1.17]
Rural (VS. urban)	1.09	[0.90,1.32]	1.13	[0.84,1.53]	1.07	[0.80,1.42]	0.99	[0.77,1.27]	1.03	[0.90,1.18]	1.05	[0.91,1.20]
Living with children (VS. alone)	1.00	[0.74,1.35]	**0.66**	**[0.46,0.95]**	1.14	[0.78,1.65]	1.08	[0.74,1.56]	**0.66**	**[0.54,0.82]**	**0.64**	**[0.52,0.79]**
Living with others, no child (VS. alone)	0.85	[0.68,1.07]	**0.58**	**[0.43,0.78]**	0.88	[0.66,1.18]	0.91	[0.69,1.20]	0.89	[0.76,1.05]	**0.72**	**[0.61,0.85]**
Close friends <3 (VS. ≥3)	**1.26**	**[1.03,1.56]**	1.18	[0.86,1.60]	0.79	[0.60,1.04]	0.97	[0.71,1.32]	0.98	[0.84,1.14]	0.89	[0.76,1.06]
Loneliness	1.00	[0.94,1.07]	**1.17**	**[1.08,1.27]**	**1.19**	**[1.10,1.28]**	**1.15**	**[1.08,1.24]**	**1.09**	**[1.04,1.14]**	1.01	[0.96,1.06]
Mental health diagnosis (VS. none)	**23.68**	**[18.82,29.81]**	**5.99**	**[4.50,7.97]**	**4.67**	**[3.42,6.38]**	**3.53**	**[2.68,4.64]**	**1.78**	**[1.50,2.11]**	**2.02**	**[1.69,2.42]**
Depression (PHQ-9)	**1.08**	**[1.05,1.11]**	**1.03**	**[1.00,1.07]**	1.03	[1.00,1.07]	**1.04**	**[1.01,1.07]**	1.01	[0.99,1.03]	**1.04**	**[1.01,1.06]**
Anxiety (GAD-7)	0.97	[0.94,1.00]	1.03	[0.99,1.07]	**1.05**	**[1.02,1.09]**	**1.06**	**[1.02,1.09]**	**1.03**	**[1.00,1.05]**	**1.05**	**[1.02,1.07]**
Coping: problem-focused	**0.78**	**[0.62,0.97]**	0.88	[0.67,1.16]	1.13	[0.87,1.47]	**1.32**	**[1.02,1.72]**	**1.98**	**[1.65,2.39]**	1.10	[0.92,1.30]
Coping: emotion-focused	1.08	[0.92,1.27]	1.16	[0.93,1.46]	1.02	[0.82,1.26]	1.21	[0.99,1.48]	**1.49**	**[1.31,1.69]**	0.99	[0.87,1.13]
Coping: avoidant	**1.30**	**[1.07,1.57]**	1.06	[0.83,1.36]	**1.30**	**[1.04,1.63]**	1.07	[0.86,1.33]	0.91	[0.78,1.05]	0.95	[0.80,1.12]
Coping: socially-supported	**1.34**	**[1.15,1.57]**	**2.14**	**[1.75,2.60]**	**1.66**	**[1.37,2.02]**	**1.58**	**[1.30,1.92]**	**1.71**	**[1.52,1.92]**	**4.18**	**[3.66,4.78]**
Personality: openness to Experience	0.95	[0.78,1.17]	1.02	[0.81,1.29]	1.04	[0.81,1.33]	0.99	[0.79,1.24]	**1.65**	**[1.43,1.90]**	1.06	[0.91,1.23]
Personality: conscientiousness	1.05	[0.89,1.24]	0.94	[0.77,1.15]	1.05	[0.86,1.28]	0.86	[0.72,1.04]	0.92	[0.81,1.04]	0.95	[0.84,1.08]
Personality: extraversion	0.90	[0.77,1.05]	1.05	[0.84,1.31]	0.96	[0.78,1.19]	1.08	[0.88,1.32]	0.92	[0.83,1.03]	**1.31**	**[1.16,1.48]**
Personality: agreeableness	1.31	[0.96,1.80]	1.01	[0.67,1.51]	1.04	[0.70,1.55]	**1.54**	**[1.03,2.29]**	1.17	[0.93,1.48]	**1.34**	**[1.04,1.71]**
Personality: neuroticism	1.07	[0.90,1.26]	1.06	[0.84,1.34]	1.10	[0.89,1.36]	0.96	[0.77,1.20]	1.08	[0.95,1.22]	**1.17**	**[1.03,1.33]**

Table S6. Results from logit model on each strategy to support mental health (no pre-existing mental health condition, N= 22,113)

	Model I: Medication		Model II: Mental health professionals		Model III: GP/other health professionals		Model IV: Helpline/online services		Model V: Self-care/help		Model VI: family/friends	
	OR	95% CI	OR	95% CI	OR	95% CI	OR	95% CI	OR	95% CI	OR	95% CI
30-45 (VS. 18-29)	1.49	[0.95,2.33]	0.91	[0.63,1.33]	0.88	[0.51,1.52]	1.07	[0.69,1.65]	0.82	[0.64,1.05]	0.80	[0.61,1.06]
46-59 (VS. 18-29)	**1.89**	**[1.21,2.96]**	0.77	[0.48,1.22]	0.63	[0.32,1.26]	0.85	[0.58,1.24]	**0.66**	**[0.51,0.85]**	**0.53**	**[0.40,0.70]**
60+ (VS. 18-29)	1.50	[0.93,2.42]	0.48	[0.23,1.02]	0.65	[0.30,1.42]	0.76	[0.49,1.15]	**0.34**	**[0.26,0.45]**	**0.32**	**[0.24,0.43]**
Women (VS. men)	1.00	[0.79,1.28]	0.97	[0.68,1.39]	0.72	[0.47,1.10]	0.97	[0.71,1.34]	**2.08**	**[1.81,2.39]**	**1.43**	**[1.24,1.65]**
Ethnic minority (VS. white)	0.81	[0.52,1.29]	1.48	[0.93,2.37]	1.49	[0.69,3.19]	**1.77**	**[1.08,2.92]**	1.06	[0.80,1.42]	0.92	[0.70,1.21]
Education medium (VS. low)	**0.76**	**[0.58,1.00]**	1.18	[0.64,2.19]	1.39	[0.84,2.32]	**1.73**	**[1.01,2.95]**	**1.32**	**[1.07,1.62]**	**1.28**	**[1.04,1.59]**
Education high (VS. low)	**0.66**	**[0.51,0.87]**	1.62	[0.96,2.72]	0.84	[0.54,1.30]	1.37	[0.78,2.39]	**2.06**	**[1.67,2.54]**	**1.48**	**[1.19,1.82]**
Employed (VS. Not employed)	1.05	[0.83,1.34]	1.14	[0.79,1.66]	0.99	[0.60,1.63]	1.27	[0.92,1.76]	1.07	[0.90,1.26]	1.18	[0.99,1.40]
Income <30k (VS. ≥30k)	**1.61**	**[1.28,2.04]**	1.04	[0.75,1.46]	1.36	[0.80,2.32]	1.06	[0.77,1.45]	0.97	[0.83,1.13]	0.93	[0.79,1.10]
Rural (VS. urban)	1.24	[1.00,1.53]	1.13	[0.78,1.66]	0.97	[0.65,1.46]	0.83	[0.61,1.13]	0.99	[0.87,1.13]	0.92	[0.80,1.06]
Living with children (VS. alone)	0.99	[0.71,1.40]	**0.63**	**[0.41,0.98]**	1.12	[0.66,1.91]	0.95	[0.63,1.44]	**0.67**	**[0.55,0.82]**	**0.75**	**[0.61,0.92]**
Living with others, no child (VS. alone)	1.14	[0.88,1.47]	0.82	[0.56,1.19]	1.10	[0.77,1.58]	1.01	[0.70,1.47]	0.92	[0.78,1.08]	**0.73**	**[0.62,0.86]**
Close friends <3 (VS. ≥3)	1.09	[0.86,1.39]	0.98	[0.62,1.54]	1.27	[0.72,2.23]	1.29	[0.85,1.94]	0.95	[0.81,1.12]	0.92	[0.77,1.09]
Loneliness	1.08	[1.00,1.16]	**1.30**	**[1.18,1.44]**	**1.20**	**[1.09,1.31]**	**1.21**	**[1.10,1.34]**	**1.09**	**[1.05,1.14]**	1.05	[1.00,1.10]
Mental health diagnosis (VS. none)	--	--	--	--	--	--	--	--	--	--	--	--
Depression (PHQ-9)	**1.07**	**[1.04,1.10]**	1.04	[1.00,1.09]	1.03	[0.99,1.07]	1.03	[0.99,1.07]	1.00	[0.98,1.03]	**1.04**	**[1.02,1.07]**
Anxiety (GAD-7)	1.00	[0.97,1.04]	1.01	[0.96,1.06]	**1.06**	**[1.02,1.11]**	1.04	[1.00,1.08]	**1.03**	**[1.01,1.06]**	**1.04**	**[1.02,1.07]**
Coping: problem-focused	0.86	[0.64,1.17]	0.77	[0.55,1.08]	**1.42**	**[1.03,1.95]**	1.36	[0.98,1.89]	**1.91**	**[1.59,2.29]**	**1.22**	**[1.03,1.45]**
Coping: emotion-focused	1.19	[0.98,1.46]	1.06	[0.79,1.40]	0.81	[0.62,1.06]	0.93	[0.66,1.30]	**1.35**	**[1.20,1.53]**	0.96	[0.84,1.09]
Coping: avoidant	**1.27**	**[1.03,1.57]**	1.10	[0.75,1.61]	1.07	[0.70,1.63]	1.06	[0.81,1.39]	**0.80**	**[0.69,0.93]**	0.89	[0.75,1.05]
Coping: socially-supported	**1.39**	**[1.14,1.68]**	**2.89**	**[2.33,3.59]**	**1.78**	**[1.29,2.45]**	**1.95**	**[1.55,2.45]**	**1.84**	**[1.64,2.06]**	**3.90**	**[3.43,4.44]**
Personality: openness to Experience	0.92	[0.75,1.14]	**1.35**	**[1.01,1.81]**	1.01	[0.72,1.41]	1.18	[0.88,1.59]	**1.75**	**[1.52,2.02]**	1.16	[1.00,1.35]
Personality: conscientiousness	0.98	[0.80,1.20]	1.03	[0.81,1.31]	1.38	[0.92,2.06]	0.99	[0.80,1.23]	0.90	[0.79,1.02]	0.95	[0.83,1.08]
Personality: extraversion	1.00	[0.84,1.18]	1.08	[0.81,1.44]	1.28	[0.91,1.81]	1.03	[0.80,1.33]	**0.84**	**[0.75,0.94]**	**1.33**	**[1.18,1.50]**
Personality: agreeableness	1.33	[0.88,2.03]	0.88	[0.51,1.49]	**0.50**	**[0.27,0.95]**	1.49	[0.93,2.38]	1.20	[0.96,1.50]	**1.30**	**[1.02,1.67]**
Personality: neuroticism	**1.36**	**[1.12,1.64]**	**1.42**	**[1.01,1.99]**	**1.42**	**[1.02,1.97]**	**1.39**	**[1.02,1.87]**	1.03	[0.92,1.16]	**1.21**	**[1.06,1.37]**

Table S7. Results from logit model on each strategy to support mental health (with pre-existing mental health condition, N=4,607)

	Model I: Medication		Model II: Mental health professionals		Model III: GP/other health professionals		Model IV: Helpline/online services		Model V: Self-care/help		Model VI: family/friends	
	OR	95% CI	OR	95% CI	OR	95% CI	OR	95% CI	OR	95% CI	OR	95% CI
30-45 (VS. 18-29)	**1.89**	**[1.31,2.73]**	0.67	[0.45,1.00]	1.14	[0.75,1.74]	1.18	[0.78,1.80]	0.87	[0.58,1.31]	0.85	[0.54,1.33]
46-59 (VS. 18-29)	**3.12**	**[2.10,4.64]**	**0.55**	**[0.36,0.83]**	1.44	[0.93,2.22]	1.15	[0.76,1.73]	**0.53**	**[0.35,0.79]**	0.82	[0.53,1.28]
60+ (VS. 18-29)	**3.45**	**[2.17,5.49]**	**0.23**	**[0.14,0.38]**	0.66	[0.40,1.09]	0.71	[0.44,1.15]	**0.34**	**[0.22,0.54]**	**0.44**	**[0.27,0.70]**
Women (VS. men)	1.12	[0.81,1.56]	0.87	[0.59,1.29]	0.93	[0.64,1.35]	0.73	[0.51,1.04]	**1.80**	**[1.35,2.40]**	1.07	[0.80,1.44]
Ethnic minority (VS. white)	**0.58**	**[0.35,0.99]**	0.93	[0.50,1.72]	0.82	[0.44,1.54]	1.62	[0.96,2.75]	0.81	[0.46,1.42]	1.14	[0.61,2.12]
Education medium (VS. low)	0.82	[0.55,1.21]	1.04	[0.68,1.59]	0.99	[0.67,1.45]	1.24	[0.85,1.81]	1.27	[0.93,1.74]	1.23	[0.86,1.77]
Education high (VS. low)	0.87	[0.59,1.28]	1.29	[0.87,1.93]	1.05	[0.71,1.54]	**1.48**	**[1.02,2.13]**	**1.76**	**[1.27,2.43]**	1.31	[0.92,1.88]
Employed (VS. Not employed)	**0.71**	**[0.53,0.96]**	0.75	[0.55,1.03]	**0.72**	**[0.52,0.99]**	0.79	[0.58,1.08]	1.11	[0.82,1.51]	1.16	[0.87,1.56]
Income <30k (VS. ≥30k)	0.86	[0.64,1.15]	**0.69**	**[0.50,0.96]**	0.87	[0.62,1.22]	1.06	[0.77,1.47]	0.97	[0.71,1.32]	1.00	[0.74,1.37]
Rural (VS. urban)	0.99	[0.69,1.42]	1.30	[0.90,1.88]	1.05	[0.76,1.46]	1.23	[0.88,1.71]	1.05	[0.78,1.42]	1.06	[0.75,1.50]
Living with children (VS. alone)	1.10	[0.74,1.65]	**0.59**	**[0.38,0.90]**	0.79	[0.51,1.22]	1.06	[0.69,1.63]	**0.62**	**[0.41,0.93]**	**0.52**	**[0.34,0.78]**
Living with others, no child (VS. alone)	0.84	[0.60,1.20]	**0.59**	**[0.42,0.83]**	0.70	[0.48,1.02]	0.93	[0.64,1.36]	0.82	[0.59,1.14]	0.76	[0.53,1.08]
Close friends <3 (VS. ≥3)	1.04	[0.77,1.42]	**1.42**	**[1.01,1.99]**	0.96	[0.69,1.34]	0.85	[0.62,1.18]	0.98	[0.74,1.28]	0.78	[0.58,1.05]
Loneliness	1.03	[0.95,1.12]	1.08	[0.99,1.18]	1.07	[0.98,1.16]	**1.11**	**[1.03,1.21]**	1.07	[0.99,1.15]	0.95	[0.87,1.03]
Mental health diagnosis (VS. none)	--	--	--	--	--	--	--	--	--	--	--	--
Depression (PHQ-9)	1.03	[1.00,1.07]	1.03	[0.99,1.07]	1.02	[0.99,1.06]	1.03	[0.99,1.07]	0.98	[0.95,1.01]	1.01	[0.98,1.05]
Anxiety (GAD-7)	0.98	[0.94,1.01]	1.02	[0.98,1.07]	**1.08**	**[1.04,1.12]**	1.05	[1.01,1.09]	1.02	[0.98,1.06]	1.05	[1.01,1.09]
Coping: problem-focused	0.76	[0.55,1.06]	0.93	[0.66,1.32]	0.94	[0.67,1.32]	1.04	[0.73,1.47]	**1.39**	**[1.02,1.89]**	0.79	[0.57,1.10]
Coping: emotion-focused	1.03	[0.81,1.32]	1.07	[0.81,1.42]	1.03	[0.79,1.32]	**1.38**	**[1.04,1.84]**	**2.03**	**[1.60,2.58]**	1.18	[0.93,1.52]
Coping: avoidant	**1.52**	**[1.18,1.97]**	1.09	[0.82,1.45]	**1.32**	**[1.02,1.72]**	1.26	[0.97,1.63]	**1.41**	**[1.11,1.80]**	1.05	[0.81,1.38]
Coping: socially-supported	1.20	[0.96,1.50]	**2.14**	**[1.68,2.75]**	**1.59**	**[1.25,2.02]**	**1.52**	**[1.19,1.93]**	**1.43**	**[1.14,1.80]**	**5.85**	**[4.50,7.60]**
Personality: openness to Experience	0.86	[0.68,1.10]	0.88	[0.67,1.14]	0.85	[0.65,1.11]	0.83	[0.65,1.07]	**1.89**	**[1.49,2.40]**	1.03	[0.80,1.33]
Personality: conscientiousness	1.10	[0.87,1.38]	0.84	[0.67,1.06]	0.94	[0.75,1.17]	0.92	[0.73,1.15]	0.90	[0.73,1.11]	0.86	[0.69,1.08]
Personality: extraversion	1.00	[0.80,1.26]	1.16	[0.92,1.46]	1.17	[0.93,1.47]	1.24	[1.00,1.53]	1.03	[0.83,1.28]	**1.52**	**[1.20,1.92]**
Personality: agreeableness	1.30	[0.84,2.02]	1.26	[0.79,2.02]	1.14	[0.73,1.79]	1.39	[0.87,2.21]	1.23	[0.80,1.90]	1.37	[0.89,2.12]
Personality: neuroticism	**0.75**	**[0.58,0.97]**	1.08	[0.79,1.47]	0.90	[0.69,1.17]	1.15	[0.89,1.48]	1.22	[0.95,1.56]	1.22	[0.95,1.56]
